# Murine cytomegalovirus infection exacerbates complex IV deficiency in a model of mitochondrial disease

**DOI:** 10.1371/journal.pgen.1008604

**Published:** 2020-03-04

**Authors:** Nicola Ferreira, Christopher E. Andoniou, Kara L. Perks, Judith A. Ermer, Danielle L. Rudler, Giulia Rossetti, Ambika Periyakaruppiah, Jamie K. Y. Wong, Oliver Rackham, Peter G. Noakes, Mariapia A. Degli-Esposti, Aleksandra Filipovska

**Affiliations:** 1 Harry Perkins Institute of Medical Research, QEII Medical Centre, Nedlands, Western Australia, Australia; 2 Centre for Medical Research, The University of Western Australia, Nedlands, Western Australia, Australia; 3 Infection and Immunity Program and Department of Microbiology, Biomedicine Discovery Institute, Monash University, Clayton, Victoria, Australia; 4 Centre for Experimental Immunology, Lions Eye Institute, Perth, Western Australia, Australia; 5 School of Biomedical Sciences, The University of Queensland, St. Lucia, Queensland, Australia; 6 School of Pharmacy and Biomedical Sciences, Curtin University, Bentley, Western Australia, Australia; 7 Curtin Health Innovation Research Institute, Curtin University, Bentley, Western Australia, Australia; 8 Telethon Kids Institute, QEII Medical Centre, Nedlands, Western Australia, Australia; 9 Queensland Brain Institute, The University of Queensland, St. Lucia, Queensland, Australia; 10 School of Molecular Sciences, The University of Western Australia, Crawley, Western Australia, Australia; University of Würzburg, GERMANY

## Abstract

The influence of environmental insults on the onset and progression of mitochondrial diseases is unknown. To evaluate the effects of infection on mitochondrial disease we used a mouse model of Leigh Syndrome, where a missense mutation in the *Taco1* gene results in the loss of the translation activator of cytochrome c oxidase subunit I (TACO1) protein. The mutation leads to an isolated complex IV deficiency that mimics the disease pathology observed in human patients with *TACO1* mutations. We infected *Taco1* mutant and wild-type mice with a murine cytomegalovirus and show that a common viral infection exacerbates the complex IV deficiency in a tissue-specific manner. We identified changes in neuromuscular morphology and tissue-specific regulation of the mammalian target of rapamycin pathway in response to viral infection. Taken together, we report for the first time that a common stress condition, such as viral infection, can exacerbate mitochondrial dysfunction in a genetic model of mitochondrial disease.

## Author summary

Mitochondrial diseases are the most commonly inherited metabolic disorders that are heterogenic and have varied disease onset and progression. Acquired infections and the associated inflammatory responses are known triggers for mitochondrial disease in the clinic and can cause progressive deterioration in patients with mitochondrial disease. Knowledge of how an infection causes and contributes to the progression of mitochondrial disease is completely lacking and has never before been investigated. Here we examined the effects of a viral infection in a model of energy dysfunction and identified that cytomegalovirus can worsen the progression of mitochondrial disease symptoms.

## Introduction

Mitochondrial diseases (MDs) are a group of progressive tissue-specific or multi-systemic disorders that are caused by defects in energy production [1]. MDs are caused by mutations in mitochondrial genes, or nuclear genes that encode mitochondrial proteins, which together constitute the most commonly inherited metabolic disorders worldwide [2]. Although not reported in the literature, in the clinic, MD patients commonly experience progressive deterioration in their clinical symptoms and suffer from profound fatigue and lethargy during infections. This effect is hypothesized to occur due to the large metabolic demand and stress imposed on mitochondria required to provide the necessary energy to overcome infection. MD patients are expected to make a full recovery following infection, however, in the case of severe infections, prolonged or reoccurring infections, patients may experience permanent regression in their clinical symptoms. It remains unknown how environmental stress conditions such as infections could potentially trigger the onset or potentiate the severity of debilitating symptoms suffered by MD patients.

Recently we developed a model of mitochondrial disease that is caused by a mutation in a nuclear gene encoding the translational activator of cytochrome c oxidase subunit 1 (TACO1). To date, TACO1 is the only known translational activator in mammalian mitochondria [3,4]. A homozygous mutation in the *TACO1* gene has been identified in patients suffering from Leigh Syndrome (LS) who have an isolated cytochrome c oxidase (or complex IV) deficiency that leads to progressive cognitive dysfunction, dystonia and visual impairment [3]. The identified *TACO1* homozygous mutation in patients is a single-base-pair insertion in position 472 (472insC) that results in a frame shift and generates a premature stop codon, which consequently causes loss of the TACO1 protein in LS patients. The TACO1 protein is a member of a large family of poorly characterised proteins that contains a 297 amino acid DUF28 domain and is imported into the mitochondrial matrix. TACO1 is composed of three domains that form a hook-like structure, domain 1 has positively charged residues that associate with the *mt-Co1* mRNA and domain 2 is required for TACO1 stability by linking domains 1 and 3 [4]. The *Taco1* mutation in mice results in the loss of the TACO1 protein and causes similar pathologies to those observed in LS patients harbouring *TACO1* mutations. Molecular defects are detectable in *Taco1* mutant mice from 4 weeks of age, with MD symptoms evident from 20 weeks of age [4], analogous to the patients who show clinical symptoms from ages 7–11 [3].

A large proportion of the human population (50–80%) harbours a latent human CMV (HCMV) infection that is typically contracted in early childhood. The CMVs are a family of species-specific viruses with murine CMV (MCMV) widely utilized as a model for HCMV infection due to the similarities in structure and biology between the two viruses [5]. We used our *Taco1* model of MD, to investigate the effects of a common viral infection on the onset and progression of MD. Here we show that infection with MCMV in a model of cytochrome oxidase c deficiency exacerbates the complex IV deficiency and mitochondrial dysfunction, causes alterations in mTOR signalling, and morphological changes in neuromuscular connections. These findings reveal that viral infection is a significant environmental modifier of genetic mitochondrial disorders and the avoidance or treatment of influencing environmental factors is vital in the management of MD.

## Results

### *Taco1* mutant mice exhibit no overt immunological defects following MCMV infection

**Fig 1:**
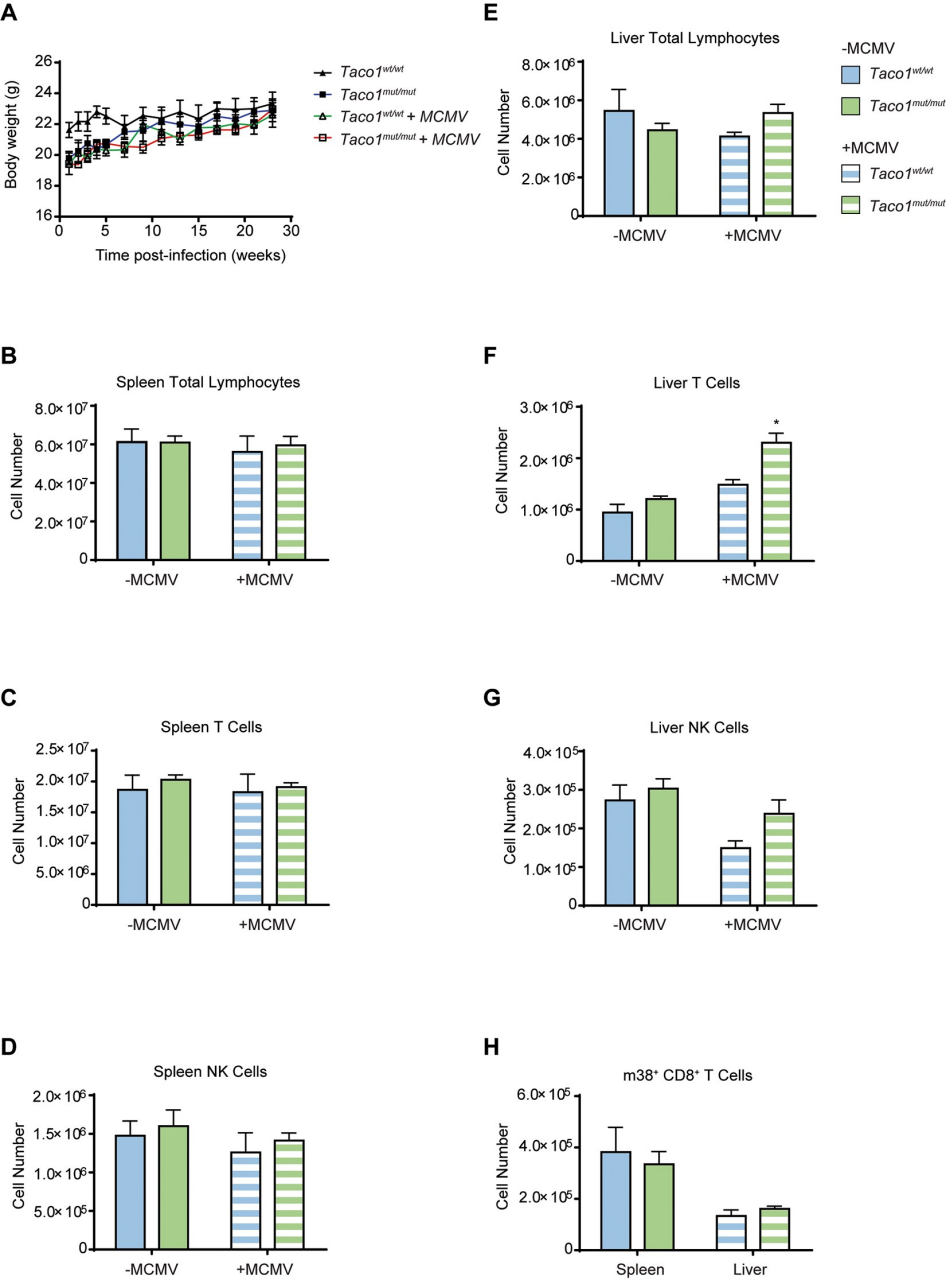
*Taco1* ^*mut/mut*^ mice generate a normal immune response. **(A)** Body weights from uninfected and MCMV infected *Taco1*^*wt/wt*^ and *Taco1* ^*mut/mut*^ mice. Spleens and livers from uninfected and MCMV infected 30 week old *Taco1*^*wt/wt*^ and *Taco1* ^*mut/mut*^ mice were prepared for flow cytometry to determine **(B)** the number of total spleen lymphocytes, **(C)** the number of spleen T cells, **(D)** the number of spleen NK cells, **(E)** the number of total liver lymphocytes, (**F)** the number of liver T cells, **(G)** the number of liver NK cells, and **(H)** the number of m38^+^ CD8^+^ T cells in the spleen and liver. The data are representative of results obtained from at least 5 mice from each genotype and each infection group. Error bars indicate SEM; *p<0.05, compared with control treatments by a two-tailed paired Student’s *t*-test.

*Taco1*^*mut/mut*^ or *Taco1*^*wt/wt*^ mice were infected with MCMV and their body weights monitored. Over the course of the experiment weight gain by MCMV-infected *Taco1*^*mut/mut*^ mice was similar to uninfected *Taco1*^*mut/mut*^ mice (Fig 1A). Furthermore, no difference in weight gain between wild-type mice and *Taco1*^*mut/mut*^ mice infected with MCMV was noted (Fig 1A). Next lymphocyte populations in the spleen and liver, two organs targeted by MCMV, were assessed at 23 weeks post-infection (p.i.) by flow cytometry. The total number of lymphocytes in the spleen and liver of *Taco1*^*mut/mut*^ was similar to that of wild-type mice, with MCMV infection having no significant impact on lymphocyte numbers (Fig 1B and 1E). T cells and natural killer (NK) cells are critical for control of MCMV infection. We did not detect a difference in the total number of these two lymphocyte populations in the spleens of *Taco1*^*mut/mut*^ mice after MCMV infection compared to controls (Fig 1C and 1D). By contrast, MCMV-infected *Taco1*^*mut/mut*^ mice had increased numbers of T cells in the livers compared to controls while NK cell numbers were not significantly different (Fig 1F and 1G). Importantly however, the number of MCMV-specific m38^+^ CD8^+^ T cells in the spleen and liver of *Taco1*^*mut/mut*^ mice was equivalent to that of *Taco1*^*wt/wt*^ mice (Fig 1H). Overall these findings indicate that *Taco1*^*mut/mut*^ mice are capable of generating an effective immune response to MCMV.

### Murine CMV infection exacerbates isolated complex IV deficiency in the heart

**Fig 2:**
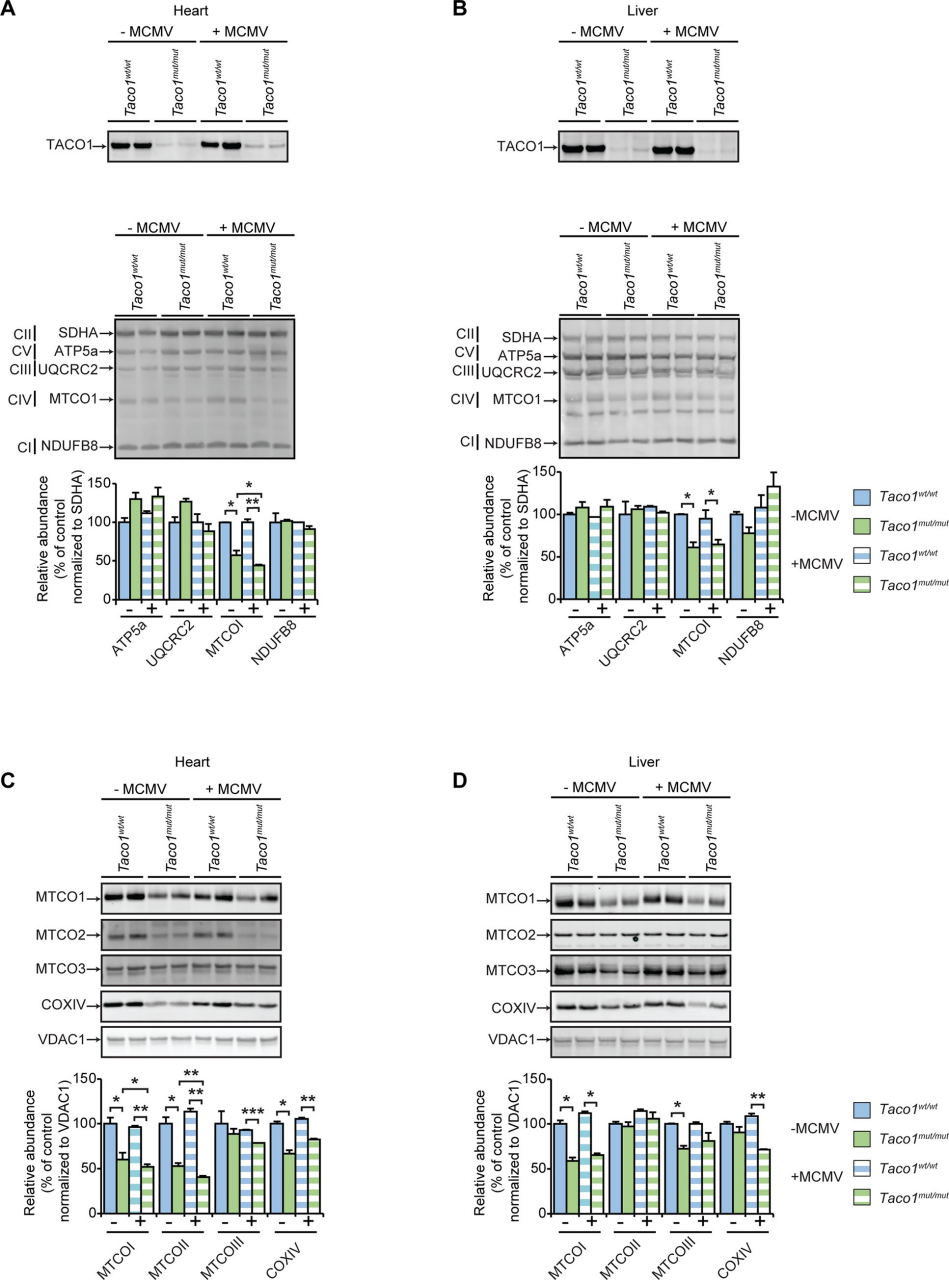
MCMV infection reduces the abundance of complex IV subunits in the heart. Homogenates from uninfected and MCMV infected 30 week old *Taco1*^*wt/wt*^ mice and *Taco1* ^*mut/mut*^ adult mice were resolved on 4–12% Bis-Tris gels and immunoblotted using antibodies to investigate the steady state levels of TACO1 and OXPHOS protein subunits in the heart (25 μg) **(A)** and in the liver (50 μg) **(B)**, as well as mitochondrial and nuclear-encoded Complex IV protein subunits in the heart **(C)** and in the liver **(D)**. CII (Succinate dehydrogenase complex subunit A (SDHA)) or VDAC1 were used as loading controls. Relative abundance of proteins was measured using Li-Cor Odyssey Classic software normalized to the loading control. All data are representative of results obtained from 4 mice of each strain and two independent biological experiments. Error bars indicate SEM; *p<0.05, **p<0.01, compared with control treatments by a two-tailed paired Student’s *t*-test.

**Fig 3:**
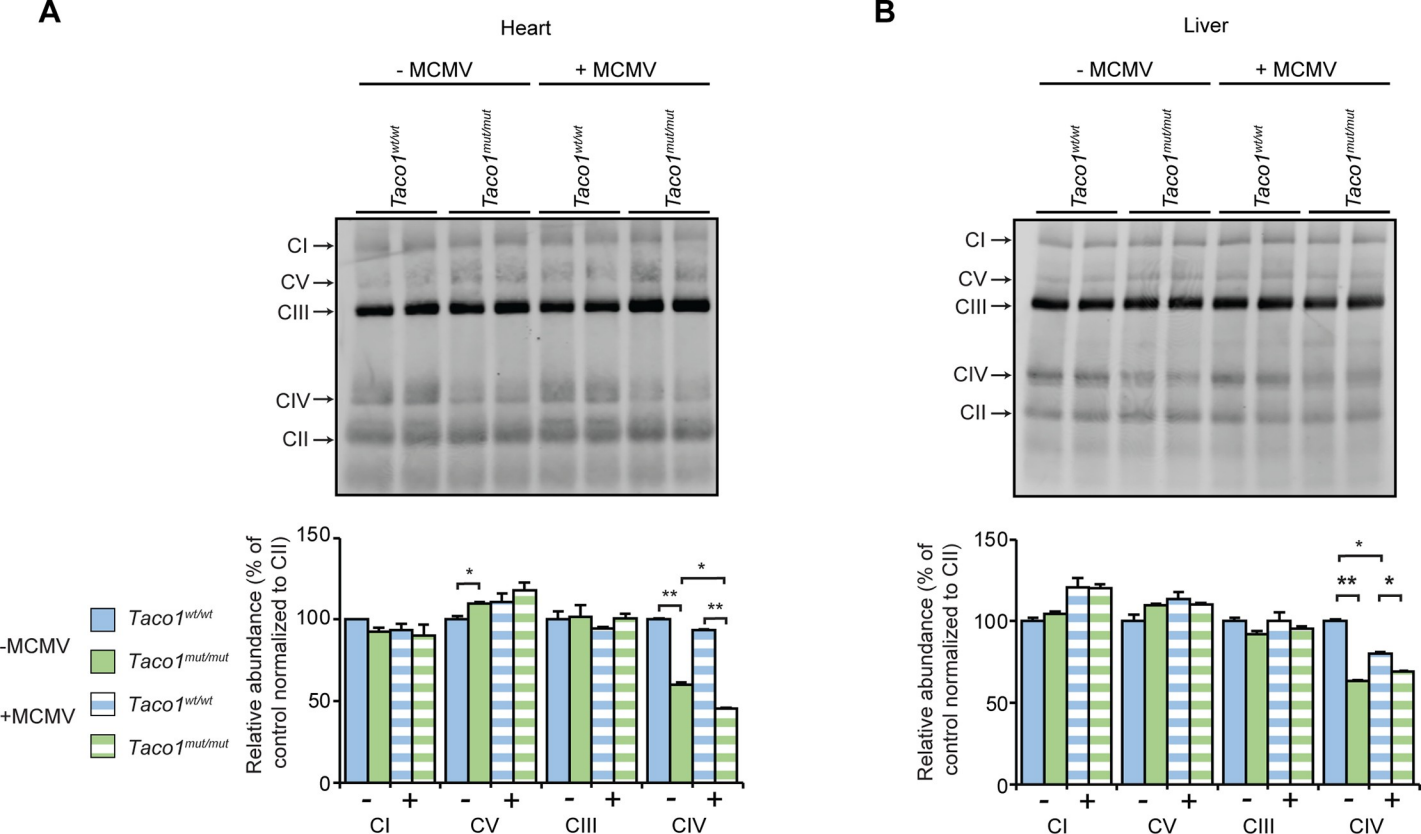
MCMV infection exacerbates mitochondrial dysfunction in the heart. **(A)** Heart homogenates (80 μg) and **(B)** liver homogenates (160 μg) from uninfected and MCMV infected 30 week old *Taco1*^*wt/wt*^ mice and *Taco1* ^*mut/mut*^ adult mice were resolved on a 4–16% BN-PAGE gels. Immunoblotting with the Blue Native OXPHOS antibody cocktail was used to visualize respiratory complexes. CII was used as a loading control. Relative abundance of proteins was measured using Li-Cor Odyssey Classic software normalized to the loading control. All data are representative of results obtained from 4 mice of each strain and two independent biological experiments. Error bars indicate SEM; *p<0.05, compared with control treatments by a two-tailed paired Student’s *t*-test.

We investigated the effects of MCMV infection on the abundance of TACO1 and mitochondrial proteins by immunoblotting in the heart and liver of 30 week old mice, and specifically the levels of cytochrome c oxidase subunit 1 (COXI) a key component of the respiratory chain that catalyses the reduction for oxygen to water, which is regulated by TACO1. The levels of TACO1 were dramatically lowered in the presence or absence of infection and there was a reduction in the abundance of COXI in the hearts and livers (Fig 2A and 2B, respectively) of *Taco1*^*mut/mut*^ mice from both groups compared to their respective control mice. This reduction of COXI was more severe in MCMV infected *Taco1*^*mut/mut*^ mice than non-infected *Taco1*^*mut/mut*^ mice in mitochondria isolated from hearts, but not in mitochondria isolated from livers. There were no differences in the abundance of protein subunits from the remaining complexes, further validating that this mutation only affects Complex IV (Fig 2A and 2B). Other mitochondrial and nuclear-encoded subunits of Complex IV were also decreased, suggesting that loss of COXI leads to decrease of other Complex IV subunits (Fig 2C and 2D) and the loss affected the mitochondrially encoded subunits in the heart more severely. Next, we analyzed the levels of the assembled respiratory complexes by blue native polyacrylamide gel electrophoresis (BN-PAGE) in heart and liver mitochondria (Fig 3A and 3B, respectively). There was a significant reduction of complex IV in the hearts of *Taco1*^*mut/mut*^ mice from both groups compared to their respective control mice (Fig 3A). This reduction of complex IV was more severe in MCMV infected *Taco1*^*mut/mut*^ mice compared to the non-infected *Taco1*^*mut/mut*^ mice, and there were no major differences in the levels of the remaining respiratory complexes in the heart (Fig 3A). Complex IV levels were reduced in both infected and non-infected livers of *Taco1*^*mut/mut*^ mice compared to control mice, however the infection did not exacerbate the decrease further (Fig 3B). The levels of the remaining respiratory complexes were not different in the livers of the infected and non-infected mutant and control mice (Fig 3B). These findings confirm the isolated complex IV deficiency previously reported [4] and show that chronic infection with MCMV exacerbates the isolated complex IV defect in heart mitochondria of *Taco1* mutant mice.

### Murine CMV causes tissue-specific changes in mTOR signalling

**Fig 4:**
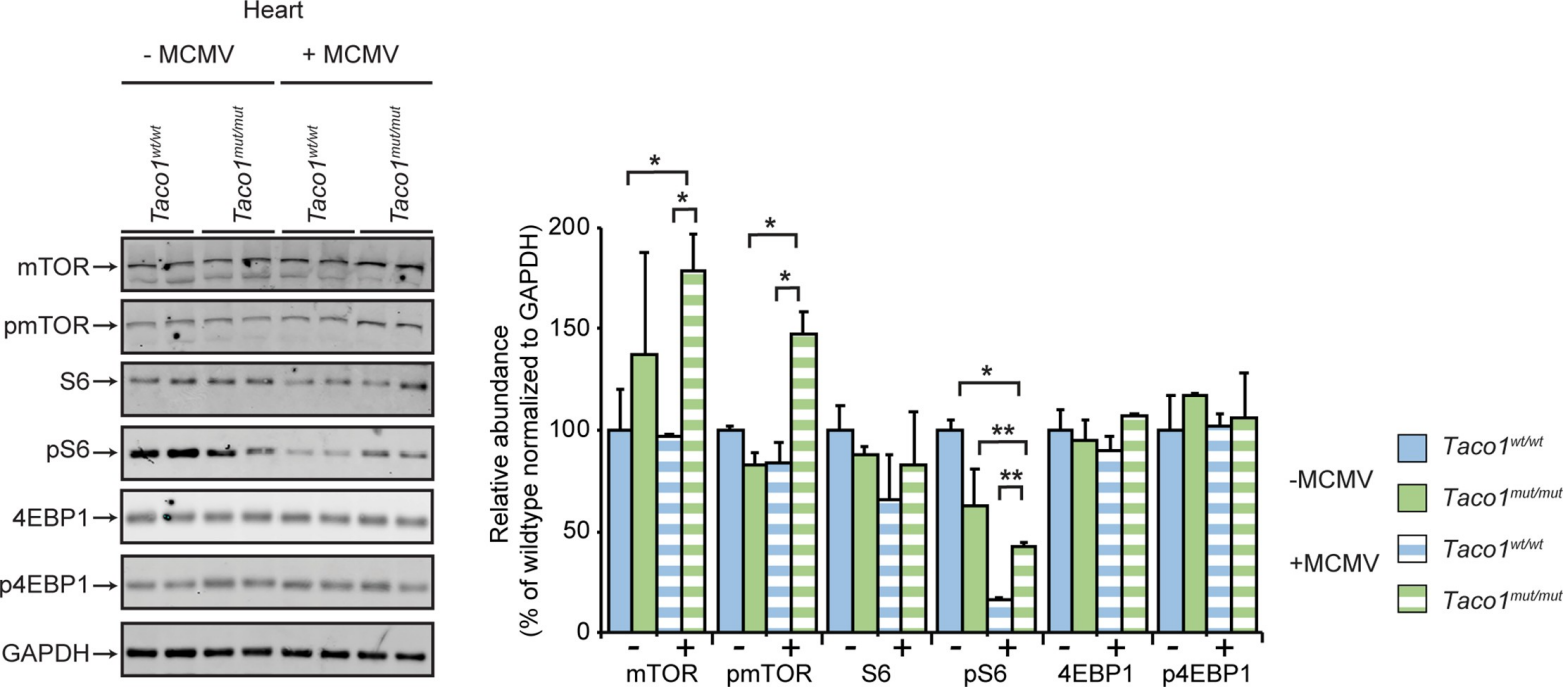
MCMV infection perturbs mTOR signalling in the heart. Heart homogenates (25 μg) from uninfected and MCMV infected 30 week old *Taco1*^*wt/wt*^ mice and *Taco1* ^*mut/mut*^ adult mice were resolved on 4–12% Bis-Tris gels and immunoblotted using antibodies to investigate the steady state levels of mTOR and its substrates. Glyceraldehyde-phosphate dehydrogenase (GAPDH) was used as a loading control. Relative abundance of proteins was measured using Li-Cor Odyssey Classic software normalized to the loading control. All data are representative of results obtained from 4 mice of each strain and two independent biological experiments. Error bars indicate SEM; *p<0.05; **p<0.01, compared with control treatments by a two-tailed paired Student’s *t*-test.

Impaired OXPHOS function has been shown to stimulate the upregulation of the mechanistic target of rapamycin (mTOR) signalling pathway [6] and mTOR activation has been implicated in cytomegalovirus infections [7,8]. Therefore, we investigated the effects of Complex IV deficiency on mTOR signalling in the presence and absence of MCMV infection. We investigated the effects of the *Taco1* mutation in response to MCMV infection on the mTOR signalling pathway by immunoblotting of heart and liver lysates isolated from uninfected and MCMV infected 30 week old mice. The levels of mTOR were not different in the hearts of uninfected *Taco1*^*mut/mut*^ and *Taco1*^*wt/wt*^ mice (Fig 4), however the levels of mTOR and phosphorylated mTOR were increased in the hearts of infected *Taco1*^*mut/mut*^ mice compared to both infected and uninfected control mice (Fig 4). The steady-state levels of S6, a downstream target of mTOR, were not altered in the uninfected *Taco1*^*mut/mut*^ and *Taco1*^*wt/wt*^ mice, whereas infection reduced the phosphorylated levels of S6 in the hearts of both control and mutant mice (Fig 4). Interestingly, despite the reduction in the phosphorylation levels of S6 in the infected control and mutant mice relative to uninfected mice, the MCMV infection caused an increase in the phosphorylated S6 levels in the hearts of *Taco1*^*mut/mut*^ mice compared to control mice (Fig 4). This increase was consistent with the increase in both the steady-state and phosphorylated mTOR levels in the hearts of infected *Taco1*^*mut/mut*^ mice compared to *Taco1*^*wt/wt*^ mice (Fig 4), indicating that increased energy demand during an infection can result in upregulation of mTOR signalling via S6 in the heart specifically. There were no differences in the levels of another mTOR substrate, 4EBP1 and phosphorylated 4EBP1, in the hearts of uninfected and infected *Taco1*^*mut/mut*^ and *Taco1*^*wt/wt*^ mice (Fig 4). These results indicate that the isolated complex IV defect combined with an infection cause upregulation of mTOR signalling in the heart.

**Fig 5:**
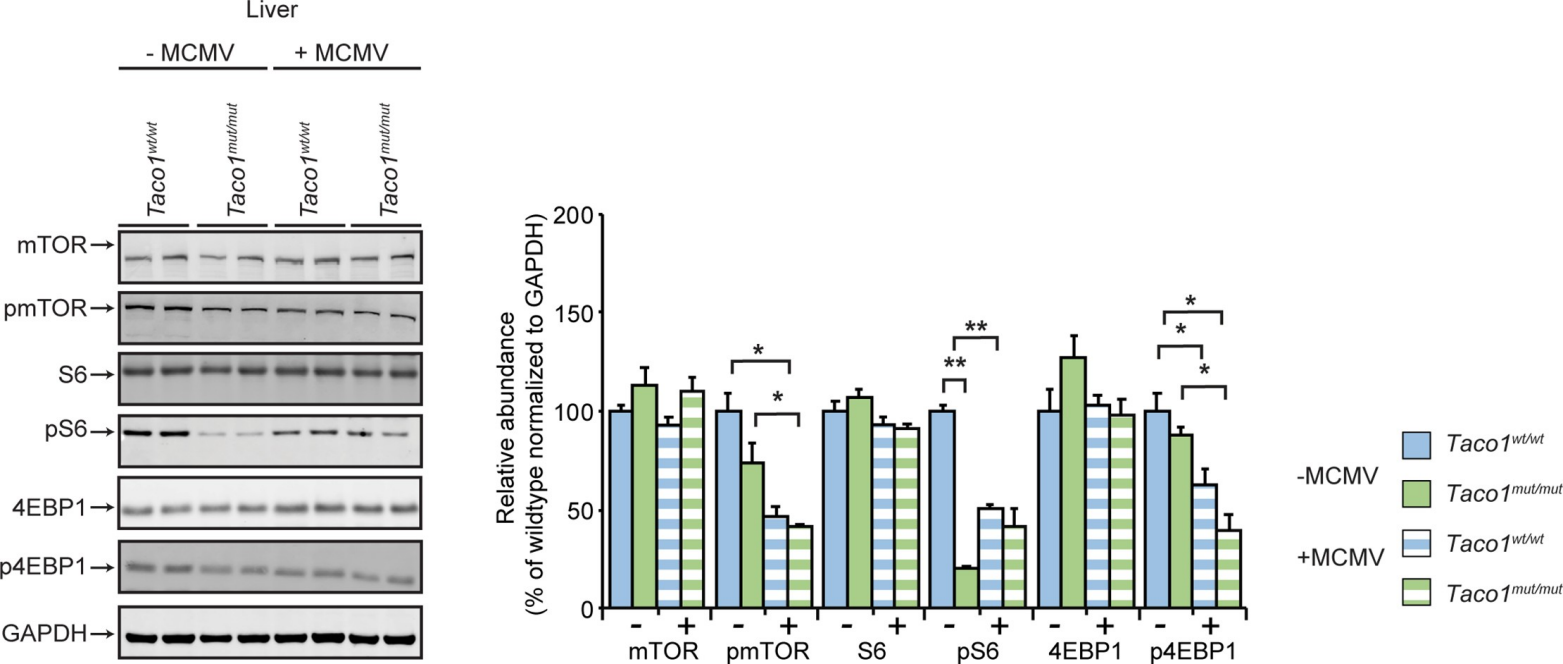
MCMV infection downregulates mTOR signalling in the liver. Liver homogenates (50 μg) from uninfected and MCMV infected 30 week old *Taco1*^*wt/wt*^ mice and *Taco1* ^*mut/mut*^ adult mice were resolved on 4–12% Bis-Tris gels and immunoblotted using antibodies to investigate the steady state levels of mTOR and its substrates. Glyceraldehyde-phosphate dehydrogenase (GAPDH) was used as a loading control. Relative abundance of proteins was measured using Li-Cor Odyssey Classic software normalized to the loading control. All data are representative of results obtained from 4 mice of each strain and two independent biological experiments. Error bars indicate SEM; *p<0.05; **p<0.01, compared with control treatments by a two-tailed paired Student’s *t*-test.

In contrast, in the livers of uninfected or MCMV infected mice, there were no differences in the steady state levels of mTOR between the *Taco1*^*mut/mut*^ and *Taco1*^*wt/wt*^ mice, whereas the levels of phosphorylated mTOR were decreased in the infected control and mutant mice compared to uninfected mice (Fig 5). The steady-state levels of S6 were not altered in the livers of uninfected *Taco1*^*mut/mut*^ and *Taco1*^*wt/wt*^ mice, however, the phosphorylated S6 levels were reduced in the uninfected mutant mice as well as the infected control and *Taco1*^*mut/mut*^ mice compared to uninfected mice (Fig 5). The increased phospho-S6 levels found in the hearts of the mutant mice were blunted in the liver and not different to the control mice (Fig 5). Consistent with decreased levels of phosphorylated mTOR and S6, the infection caused reduction in the phosphorylated levels of 4EBP1 in the livers of the control mice relative to uninfected control mice as well as in infected mutant mice relative to uninfected control and mutant mice (Fig 5). These findings indicate that in the liver an isolated complex IV defect leads to downregulation of mTOR signalling and consequently reduced S6 and 4EBP1 phosphorylation.

Taken together, these results show tissue-specific regulation of the mTOR signalling pathway in mitochondrial disease that depends on the energy requirements of individual tissues. In the heart mTOR signalling is upregulated upon infection, indicating that additional energy impost on the mutant mice activates the mTOR stress response in the heart, while in the liver mTOR signalling is downregulated.

### Chronic murine CMV infection results in reduced Complex IV activity

**Fig 6:**
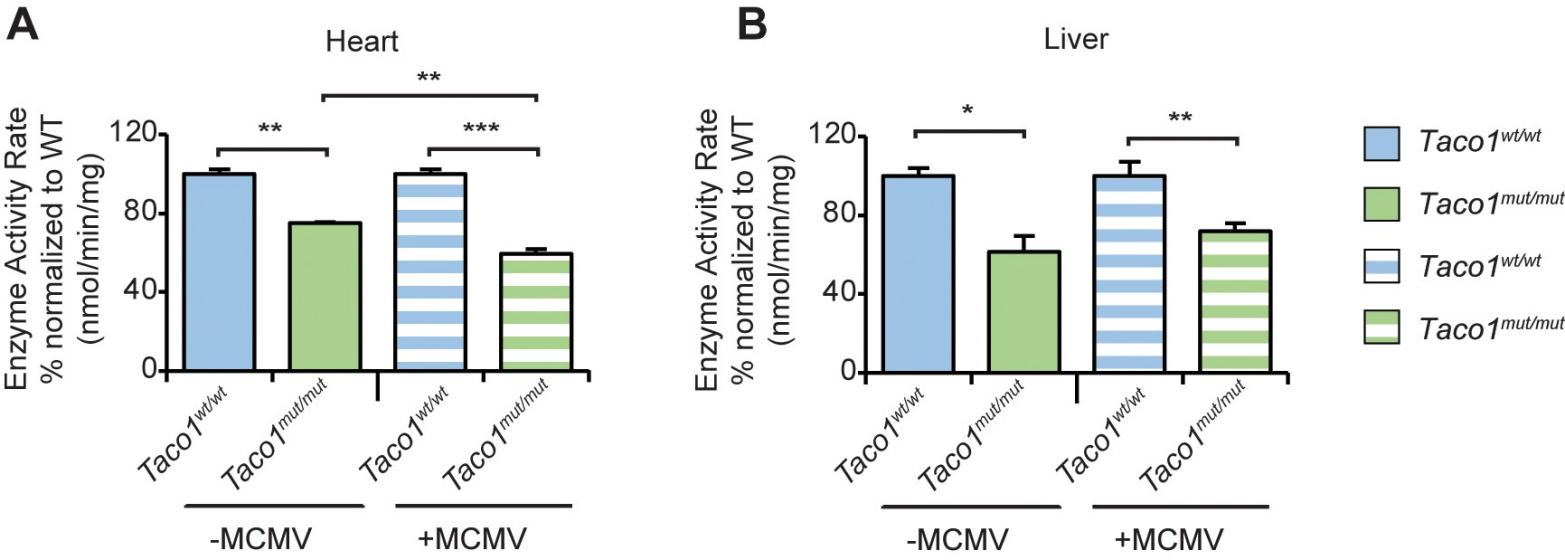
Complex IV activity is exacerbated in the hearts of *Taco1* ^*mut/mut*^ mice infected with MCMV. Complex IV activity was measured in heart **(A)** and liver **(B)** mitochondria from 30 week old uninfected and MCMV infected *Taco1*^*wt/wt*^ and *Taco1* ^*mut/mut*^ mice. Complex IV activity was measured spectrophotometrically as nmol/min/mg of protein and enzyme activity is shown as percent of activity in control mice. Error bars indicate SEM of 5 mice per genotype and treatment; *p<0.05; **p<0.01; ***p<0.001, compared with controls by a two-tailed paired Student’s *t*-test.

To determine the effects of chronic MCMV infection in the hearts and livers of control and mutant mice on mitochondrial function, we measured Complex IV activity in uninfected and MCMV infected 30 week old mice. Complex IV activity was significantly reduced in both infected and uninfected mutant mice compared to control mice (Fig 6A). In addition, the MCMV infection caused a greater decrease in Complex IV activity in the hearts of infected compared to uninfected *Taco1*^*mut/mut*^ mice (Fig 6A), indicating that MCMV infection can exacerbate mitochondrial dysfunction. In the liver complex IV activity was also significantly reduced in both infected and uninfected mutant mice compared to control mice (Fig 6B), however, infection did not exacerbate this dysfunction. We measured Complex IV activity in the hearts and livers of 9 week old mice both infected (day 4 p.i.) and uninfected *Taco1*^*mut/mut*^ mice and *Taco1*^*wt/wt*^ mice (S1A Fig). The *Taco1* mutation reduced Complex IV activity and this reduction was not further exacerbated by the infection indicating that chronic viral infection can further promote the Complex IV deficiency. Oxygen consumption measurements in liver mitochondria of uninfected and MCMV infected 9 week old mice, validate these findings, where uninfected and infected *Taco1*^*mut/mut*^ mice have an overall reduced oxygen consumption rate with reduced basal respiration, ATP production, proton leak, maximal respiration, non-mitochondrial respiration and spare capacity compared to *Taco1*^*wt/wt*^ mice (S1B Fig). This shows that energy production is impaired as a consequence of the *Taco1* mutation, as it has been observed previously [4]. Interestingly, in the infected mice, we also found that *Taco1*^*mut/mut*^ mice had increased proton leak compared to control mice and uninfected mutant mice (S1 Fig) indicating that MCMV infection exacerbates the damage to the mitochondrial membranes of the mutant mice.

### Murine CMV infection causes motor axon thinning of neuromuscular junctions in skeletal muscle

**Fig 7:**
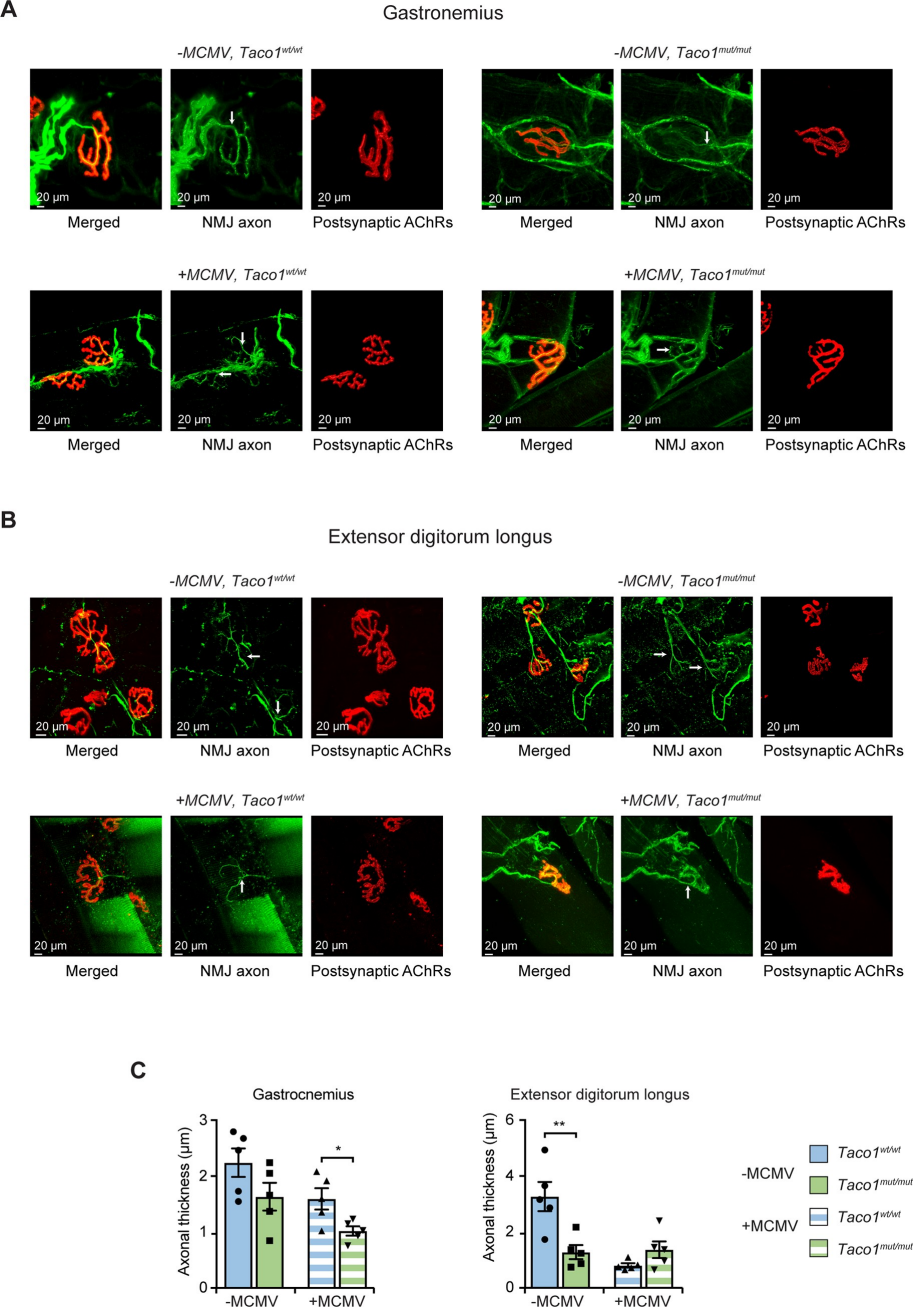
Mutation in *Taco1* along with MCMV infection causes NMJ-axonal thinning in skeletal muscle. Shown are sampled NMJs that have been stained for motor axons and their terminal endings (NMJ axonal profiles) that overlie postsynaptic acetylcholine receptors (AChRs). For each NMJ, there is a set of three images: the first is the merge of NMJ axons and their terminal ends (green) overlying postsynaptic AChRs (red), followed by single channel images of NMJ axons (green) and postsynaptic AChRs (red). Arrows indicate the motor axon entry point of each NMJ. (**A)** Shown are representative confocal micrographs of NMJs of the gastrocnemius from uninfected and infected *Taco1*^*wt/wt*^ and *Taco1*^*mut/mut*^ mice. (**B**) Representative confocal micrographs of NMJs of the extensor digitorum longus (EDL) muscle from uninfected and chronically infected *Taco1*^*wt/wt*^ and *Taco1* ^*mut/mut*^ mice. All NMJs were imaged from intact skeletal muscles (whole mount immuno-stained samples). **(C)** NMJ axonal thickness at the NMJ entry point (indicated by the arrows in the NMJ panels). NMJ axonal diameter was measured from five mice of each genotype and infection cohort (non-MCMV infected [-MCMV]; and MCMV infected [+MCMV]). Error bars indicate SEM; *p<0.05; **p<0.01, compared with control treatments by a two-tailed paired Student’s *t*-test. Scale bars = 20μm.

Deterioration of neuromuscular junctions (NMJs) has been observed following mitochondrial dysfunction in a mouse model of amyotrophic lateral sclerosis [9]. To investigate if changes occur in the morphology of the neurons and their synaptic connections, we analyzed skeletal NMJs by immunofluorescent labelling, in uninfected and infected 30 week old *Taco1*^*mut/mut*^ and *Taco1*^*wt/wt*^ mice. Neuromuscular junctions were stained using antibodies against neurofilaments and synaptic vesicles to label the motor axons and their terminal endings (pre-synaptic components), together with labelled α-bungarotoxin to identify post-synaptic acetylcholine receptors. Confocal imaging of the gastrocnemius showed a significant reduction in motor axon thickness projecting to NMJs in *Taco1*^*mut/mut*^ mice compared to *Taco1*^*wt/wt*^ mice in both infected and uninfected groups (Fig 7A and 7C). Examination of the extensor digitorum longus, which is a predominantly fast twitch muscle, showed a similar significant reduction in NMJ axonal thickness in *Taco1*^*mut/mut*^ mice compared to *Taco1*^*wt/wt*^ mice for the uninfected cohort (Fig 7B). While this reduction in axonal thickness was further reduced at NMJs from MCMV infected *Taco1*^*wt/wt*^ mice compared to uninfected *Taco1*^*wt/wt*^ and *Taco1*^*mut/mut*^ mice, there was no further decrease in NMJ axonal thickness at NMJs from infected *Taco1*^*mut/mut*^ mice (Fig 7B and 7C). These observations suggest that the mutation in *Taco1* causes morphological changes to the NMJ axons in the gastrocnemius and extensor digitorum longus muscles. While the presence of MCMV infection did cause further reductions in NMJ axonal thickness for NMJs of the gastrocnemius muscle, it did not exacerbate the reduction in NMJ axonal thickness of EDL NMJs from *Taco*^*mut/mut*^ mice. Taken together these findings suggest that MCMV infection can cause alterations in neuromuscular axonal morphology, that only intensify the phenotype in a muscle-type specific manner.

## Discussion

We used a mouse model of late-onset Leigh Syndrome to investigate how a common viral infection can trigger the onset or progression of mitochondrial disease. We identified that infection with murine cytomegalovirus does not trigger an earlier onset of the disease but instead exacerbates the complex IV deficiency and causes alterations in mTOR signalling, energy metabolism and neuromuscular morphology. Cytomegalovirus is a common species-specific virus that belongs to the herpesvirus family. Infection with cytomegalovirus places a significant metabolic stress on a host cell due to the increased biosynthesis and energy utilization required for viral replication. Here we show that MCMV infection poses a significant stress on the heart in *Taco1* mutant mice as mitochondrial biogenesis is severely impaired in the heart compared to the liver. It is possible that the infection imposes a greater cost on mitochondrial function in the heart because it predominantly relies on OXPHOS to maintain sufficient ATP levels [10]. Similar molecular defects were identified in the liver and viral infection increased the proton leak in the mutant mouse livers. However, the liver was able to cope with the additional energy demand, likely by using the spare respiratory capacity that may compensate to maintain mitochondrial function. We suggest that the ability of organs to use different metabolic and signalling pathways accounts for the tissue-specific molecular effects observed during viral infection. This differential ability of tissues to respond to mitochondrial stress has been observed recently in another model, where mitochondrial dysfunction caused by error-prone mitochondrial translation could recover due to liver-specific signalling that stimulated cellular proliferation and mitochondrial biogenesis [11]. It has also been demonstrated that human cytomegalovirus can itself disrupt cellular metabolism to cause metabolic reprogramming with an increase in biosynthetic reactions and glycolysis to maintain ATP levels for viral replication [12–14].

In the presence of infection, we identified downregulation of the mTOR signalling pathway via its downstream targets, S6 and 4EBP1, in the livers of *Taco1* mutant mice. However, MCMV infection upregulated mTOR signalling through the phosphorylation of both mTOR and S6 in the hearts of *Taco1* mutant mice. Upregulation in mTORC1 signalling has been observed previously in mitochondrial dysfunction that increases cytoplasmic protein synthesis in an effort to compensate for the severe OXPHOS defects [15,16]. This coincides with our findings where MCMV infection severely impairs mitochondrial biogenesis, particularly in heart mitochondria, that likely activates mTOR signalling in an attempt to overcome the OXPHOS defect, while the liver could maintain required ATP levels via glycolysis or gluconeogenesis and instead downregulates mTOR signalling, in an effort to preserve energy.

During infection cellular stress responses are activated that normally inhibit mTOR activation, which may act to preserve energy expenditure and prevent cytoplasmic translation of viral proteins [17,18]. However human cytomegalovirus has been shown to overcome these stress responses to activate mTOR signalling and maintain translation of viral proteins in *in vitro* studies [7,8,19]. These inconsistencies in mTOR signalling in response to viral infection may be attributed to the duration and stage of infection and species-specific effects. Furthermore, it still remains poorly understood how cytomegaloviruses can interact with a host cell to alter mTOR signalling and the consequences in different tissues.

Here we show for the first time that loss of TACO1 results in the development of significant neuromotor degeneration in skeletal muscle, exemplified by motor axon thinning including their terminal ends. This NMJ associated axonal thinning may contribute to the development of neurological symptoms that have been documented previously in this mutant mouse such as a loss of muscle grip strength and poor learning performance [4], which is commonly found in Leigh Syndrome patients [3]. In addition, we show that infection with cytomegalovirus exacerbates neurodegeneration. Little is known about the effects of human CMV on motor axons innervating skeletal muscle, however it is known that human CMV can alter the structure of CNS neurons including the retraction/alignment of their dendritic arbors [20], which along with effects on neural stem cells and progenitor cells in the brain, can result in brain disorders (reviewed in [21]). Pseudorabies virus and herpes simplex virus type 1 (HSV-1) infections have been shown to disrupt mitochondrial motility and morphology in the superior cervical ganglion (SCG) neurons of rodents by modulating mitochondrial proteins [22]. Our study extends this possible link where MCMV could also have the capacity to invade the nervous system and may have similar effects on mitochondrial function within axons, exacerbating the defects caused by reduced levels of Complex IV. We therefore provide a potential pathogenic link between the development of mitochondrial disease and the onset of neurological symptoms in Leigh Syndrome patients.

The characterization of this model of late-onset mitochondrial disease has provided insight into the contribution of acquired infections to the progression of mitochondrial disease. This study established that an environmental stress such as a viral infection can play a significant role in disease pathogenesis, neuromuscular morphology and the molecular defects caused by a genetic mutation. Infections can be potent modifiers of energy metabolism with the ability to regulate mTOR signalling. Future studies will focus on understanding the molecular and pathophysiological mechanisms in which environmental stresses impact mitochondrial disease and to establish treatments to alleviate clinical symptoms of both the infection and mitochondrial disease.

## Methods

### Animals and housing

Female wild-type (*Taco1*^*wt/wt*^) and homozygous mutant mice (*Taco1*^*mut/mut*^) carrying an ENU-induced point mutation, T491A in the *Taco1* gene, were bred onto a C57Bl/6J background for 10–12 generations. Animals were housed in standard cages (45 cm Å~ 29 cm Å~ 12 cm) under a 12-h light/dark schedule (lights on 7 a.m. to 7 p.m.) in controlled environmental conditions of 22 + 2°C and 50 + 10% relative humidity. Normal chow diet with physiological levels of thiamine (Rat & Mouse Chow, Specialty Foods, Glen Forrest, Western Australia) and water were provided ad libitum.

### Ethics statement

The study was approved by the Animal Ethics Committee of the UWA and the Harry Perkins Institute of Medical Research Animal Ethics Committee (approved protocol numbers: RA/3/100/1256, RA/3/300/98, AE031/2015 and RA/3/300/125) and was performed in accordance with Principles of Laboratory Care (NHMRC Australian code for the care and use of animals for scientific purposes, 8th Edition 2013).

### Viral infection

Mice were infected by intraperitoneal administration of 1 x 10^4^ plaque-forming units (pfu) of salivary gland propagated MCMV-K181-Perth [23].

### Flow cytometry

Spleens were passed through a steel mesh in order to generate a single cell-suspension. Single- cell suspensions from the liver were prepared by perfusing the liver via the portal vein with phosphate buffered saline. The liver was then passed through a steel mesh. The resulting preparation was resuspended in a 37.5% isotonic Percoll solution (Pharmacia) and centrifuged at 690 *g* for 12 min to separate lymphocytes from hepatocytes. Red blood cells were osmotically lysed using NH_4_Cl and cells washed in FACS buffer. The resulting single cell preparations were stained with antibodies specific for CD11b (M1/70), CD3 (145-2C11), CD4 (RM4-5), CD8 (53–6.7), NK1.1 (PK136), CD11c (HL3), CD19 (6D5). Virus-specific CD8^+^ T cells were identified using m38 tetramer (H-2K^b^-SSPPMFRV) from Immuno ID Tetramers (Melbourne, Victoria). Antibodies were obtained from BD Biosciences, BioLegend, or eBioscience. Fixable viability stain 620 (BD Biosciences) was used for live/dead discrimination. Samples were run on a LSRFortessa X-20 instrument (BD Biosciences) and results were analyzed using FlowJo software (TreeStar).

### Wholemount immunohistochemistry

Mice were anaesthetized with carbon dioxide and then euthanized by cervical dislocation. A minimum of five animals was used for each genetic and MCMV infection group. The gastrocnemius and extensor digitorium muscles were then dissected from the lower legs of these mice, pinned out at resting muscle length and fixed in 4% paraformaldehyde (PFA) in PBS for approximately 60 min at room temperature. They were then placed into 0.1M glycine in PBS for several hours, followed by washing in PBS. Small muscle bundles were dissected from these muscles and processed for whole mount immunostaining. In brief, muscle were incubated with 4% bovine serum albumin (BSA) 0.4% triton-X100 (TX-100) in PBS (blocking buffer) for 1 h at room temperature. For primary antibody staining, muscles were incubated in rabbit anti-neurofilament 1:500 and mouse anti-synaptic vesicle in 2% BSA 0.4% TX-100 in PBS overnight at 4°C. The muscles were then washed three times with PBS for 10 min prior to incubation with secondary antibody 1:1000 goat anti-rabbit Alexa 488, 1:1000 goat anti-mouse Alexa 488 and co-stained for acetylcholine receptors at the NMJ with 1:1000 Alexa 555-αBTX in PBS. Muscles were then washed with PBS and mounted in Prolong Gold antifade reagent (Molecular Probes, Invitrogen). The motor axons and their NMJs were imaged using a Leica SP8 confocal. Images were taken at a resolution of 1024 by 1024 pixels in *xy*, using a 63x Oil objective with a z-step size of 0.3 μm, providing a voxel size of 0.124 x 0.124 x 0.3 μm. Images were captured using identical laser power levels, photomultiplier gain levels, scanning speed and pinhole size. Z-stacked images were projected into a 2D rendered image to show the top down view of NMJs and their innervating motor axons. The diameter of preterminal axons before the entry of motor axon into the nerve terminal region at each NMJ was determined using Imaris 8.1.2 software (Bitplane, South Windsor, CT, USA). Collected images were assembled in Adobe Photoshop and Adobe Illustrator (Adobe Inc. USA).

### Heart and liver homogenate preparation

Heart and liver tissue were homogenized in 100 μl of 100 mM Tris, 2 mM Na3VO4, 100 mM NaCl, 1% Triton X-100, 1 mM EDTA, 10% Glycerol, 1 mM EGTA, 0.1% SDS, 1 mM NaF, 0.5% deoxycholate, 20mM Na4P2O7, pH 7.4 containing PhosSTOP Phosphatase Inhibitor Cocktail (Roche) and EDTA-free Complete protease inhibitor cocktail (Roche). The homogenate was centrifuged at 10,000 *g* for 5 min at 4°C. The previous steps were repeated until a clear tissue homogenate was produced. The tissue homogenate protein concentration was quantified using the bicinchoninic acid (BCA) assay using bovine serum albumin (BSA) as a standard.

### Mitochondrial isolation

Mitochondria were collected from homogenized livers and isolated by differential centrifugation as described previously [10], with some modifications. Livers were homogenized in buffer containing 250 mM sucrose, 5 mM Tris, 1mM EGTA, pH 7.4 with EDTA-free Complete protease inhibitor cocktail (Roche) before differential centrifugation. The mitochondrial protein concentration was quantified using the BCA assay using BSA as a standard.

### Immunoblotting

Specific proteins were detected using mouse monoclonal antibodies against SDHA (ab14715), total OXPHOS cocktail (Abcam, ab110413), total OXPHOS blue native cocktail (Abcam, ab110412) (Diluted 1:1000). Specific rabbit monoclonal antibodies were used against: S6 ribosomal protein (5G10) (CST, 2217), phospho-S6 ribosomal protein (Ser235/236) (2F9) (CST, 4856), mTOR (7C10) (CST, 2983), phospho-mTOR (Ser2448) (D9C2) (CST, 5536), 4E-BP1 (53H11) (CST, 9644) and phospho-4E-BP1 (Thr37/46) (236B4) (CST, 2855) (Diluted 1:500). IR Dye 800CW Goat Anti-Rabbit IgG or IRDye 680LT Goat Anti-Mouse IgG (Li-Cor) secondary antibodies were used and the immunoblots were visualized using an Odyssey Infrared Imaging System (Li-Cor).

### BN-PAGE

Heart tissue was homogenized in 100 μl of 0.75 M aminocaproic acid, 50 mM BisTris-HCl, pH 7.0, and 1.2% n-dodecyl -D-maltoside (DDM) containing EDTA-free Complete protease inhibitor cocktail (Roche). The homogenate was centrifuged at 10,000 *g* for 5 min at 4°C. The previous steps were repeated until a clear tissue homogenate was produced. The tissue homogenate protein concentration was quantified using the BCA assay using BSA as a standard. BN-PAGE was performed in 4–16% gradient gels according to recommendation of the Novex NativePAGE Bis-Tris gel System. PVDF membranes were used to transfer proteins from native gels followed by immunoblotting.

### Complex IV measurements

Enzyme assays were carried out in a 1-ml cuvette at 30˚C using a PerkinElmer lambda 35 dual beam spectrophotometer. Complex IV was measured as the cyanide-sensitive oxidation of ferrocytochrome c as described before [4].

### Mitochondrial respiration measurements

Mitochondrial oxygen consumption was measured in isolated liver mitochondria using the Oxygraph 2k respirometer (Oroboros Instruments) to measure the oxygen consumption rate in Mir05 buffer containing EGTA (0.5 mM), MgCl_2_.6H_2_0 (3 mM), lactobionic acid (60 mM), taurine (20 mM), KH_2_PO_4_ (10 mM), HEPES (20 mM), D-Sucrose (110 mM) and BSA, and essential fatty acid free (1 g/L). The pH was adjusted to pH 7.1. Oxygen consumption was plotted from 3 time points over a 12 minute period measuring basal respiration (10 mM Glutamate, 2 mM Malate, 5 mM Pyruvate), proton leak (0.5 μM Oligomycin), maximal respiration (0.5 μM to 1 mM FCCP) and non-mitochondrial respiration (0.5 μM Rotenone and 2.5 μM Antimycin). ATP production and reserve capacity were extrapolated from graphed data.

### Statistical analysis

For statistical analysis a two-tailed Student’s *t*-test with replicates was performed using statistical software (Excel, 2016). Numerical data for specific figures are in S1 Table.

## Supporting information

S1 Fig Oxygen consumption in mitochondria isolated from hearts and livers of *Taco^wt/wt^* and *Taco^mut/mut^* mice. **(A)** Complex IV activity was measured in heart and liver mitochondria from 9 week old uninfected and MCMV infected *Taco1*^*wt/wt*^ and *Taco1* ^*mut/mut*^ mice. Complex IV activity was measured spectrophotometrically as nmol/min/mg of protein and enzyme activity is shown as percent of activity in control mice. **(B)** Oxygen consumption was measured in liver mitochondria from 9 week old uninfected *Taco1*^*wt/wt*^ mice and *Taco1* ^*mut/mut*^ and MCMV infected 9 week old *Taco1*^*wt/wt*^ mice and *Taco1* ^*mut/mut*^ using an Oroboros oxygen electrode using glutamate, malate and pyruvate as substrates under basal conditions followed by the sequential addition of rotenone, antimycin, oligomycin and FCCP. All data are representative of results obtained from 5 mice of each strain. R = routine/basal respiration, L = proton leak, ET-capacity = maximal respiration, ROX = residual oxygen. Error bars indicate SEM of 5 mice per genotype and treatment; *p<0.05; **p<0.01; ***p<0.001, compared with controls by a two-tailed paired Student’s *t*-test.
(TIF)

S1 Table Numerical data for individual figures. (XLSX)
