# Supplementary figures and images for: Murine cytomegalovirus infection exacerbates complex IV deficiency in a model of mitochondrial disease
Source: PLoS Genet. 2020 Mar 4;16(3):e1008604. doi: 10.1371/journal.pgen.1008604 (PMC7055822; doi:10.1371/journal.pgen.1008604)

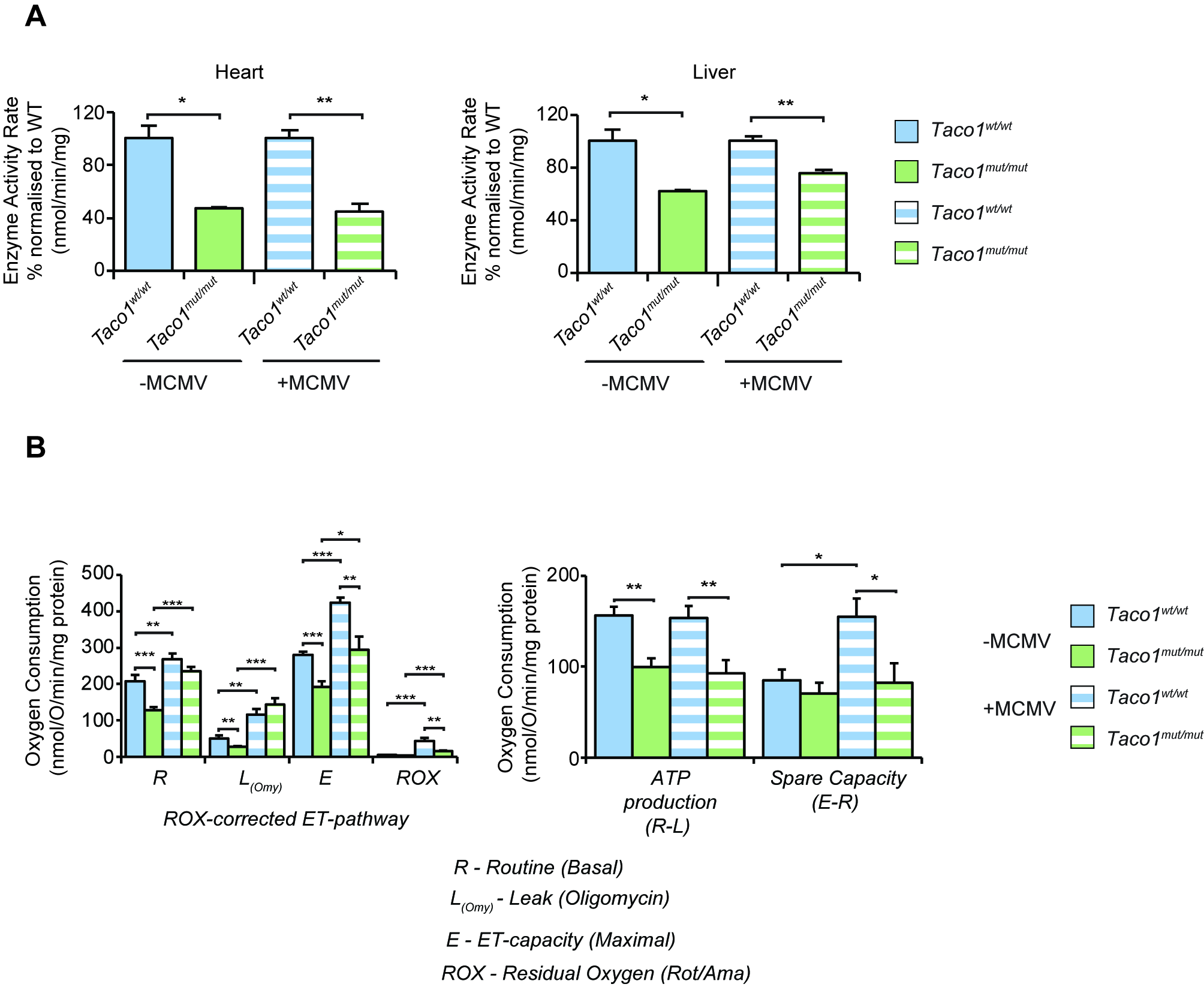

Supplement: S1 Fig — (A) Complex IV activity was measured in heart and liver mitochondria from 9 week old uninfected and MCMV infected Taco1wt/wt and Taco1 mut/mut mice. Complex IV activity was measured spectrophotometrically as nmol/min/mg of protein and enzyme activity is shown as percent of activity in control mice. (B) Oxygen consumption was measured in liver mitochondria from 9 week old uninfected Taco1wt/wt mice and Taco1 mut/mut and MCMV infected 9 week old Taco1wt/wt mice and Taco1 mut/mut using an Oroboros oxygen electrode using glutamate, malate and pyruvate as substrates under basal conditions followed by the sequential addition of rotenone, antimycin, oligomycin and FCCP. All data are representative of results obtained from 5 mice of each strain. R = routine/basal respiration, L = proton leak, ET-capacity = maximal respiration, ROX = residual oxygen. Error bars indicate SEM of 5 mice per genotype and treatment; *p<0.05; **p<0.01; ***p<0.001, compared with controls by a two-tailed paired Student’s t-test. (TIF) [file pgen.1008604.s001.tif]
